# From early markers to neuro-developmental mechanisms of autism

**DOI:** 10.1016/j.dr.2014.05.003

**Published:** 2014-09

**Authors:** T. Gliga, E.J.H. Jones, R. Bedford, T. Charman, M.H. Johnson

**Affiliations:** a Centre for Brain and Cognitive Development, Birkbeck College, University of London, United Kingdom; b Biostatistics Department, Institute of Psychiatry, King’s College London, United Kingdom; c Psychology Department, Institute of Psychiatry, King’s College London, United Kingdom

**Keywords:** Infants, Autism, The “social brain”, Sensory processing

## Abstract

A fast growing field, the study of infants at risk because of having an older sibling with autism (i.e. infant sibs) aims to identify the earliest signs of this disorder, which would allow for earlier diagnosis and intervention. More importantly, we argue, these studies offer the opportunity to validate existing neuro-developmental models of autism against experimental evidence. Although autism is mainly seen as a disorder of social interaction and communication, emerging early markers do not exclusively reflect impairments of the “social brain”. Evidence for atypical development of sensory and attentional systems highlight the need to move away from localized deficits to models suggesting brain-wide involvement in autism pathology. We discuss the implications infant sibs findings have for future work into the biology of autism and the development of interventions.

## Highlights

• Studies of infants at-risk could reveal the developmental origin of autism.
• Behavioral and brain markers differentiate infants that develop autism symptoms from controls, during the first year of life.
• Little evidence for decreased social orienting or social motivation.
• Some evidence for multiple developmental pathways to autism.

## Introduction

Autism is a disorder of social interaction and communication skills, accompanied by a restricted repertoire of interests and behaviors and by atypical sensory reactivity (DSM-5, American Psychiatric Association, 2013). In terms of behavioral signs, autism emerges over the first few years of life but robust clinical diagnosis is not typically achieved before 3 years of age (Steiner, Goldsmith, & Snow, 2012). It is therefore highly likely that, by the age of diagnosis, the symptoms presenting will be a result not only of early neurodevelopmental atypicalities, but also of adaptations and compounded effects that result from a child developing atypically within their social and physical environment for several years. A child’s decreased responsivity or seeking of social interaction, for example, could discourage others from offering the right amount and quality of social input and thus further decrease social learning. Alternatively, social isolation may act as a protective mechanism (an adaptation) for an organism overwhelmed with the richness of the input one is exposed to in social interaction (Johnson, Jones, & Gliga, in press ). Interactions between neural systems during development will also make it difficult to isolate primary and secondary causes. Decreased cortical specialization for face processing, for example, could result from decreased specialization of cortical circuitry, or alternatively, from downstream disturbance in subcortical structures driving environmental exposure to faces, early in life. Moreover, primary impairments could be transitory, and thus impossible to measure later in life, whilst still having knock-on effects on later development.

While some attempts are being made to differentiate compensations and compounded effects from the primary deficit using neuroimaging (Kaiser et al., 2010), it is evident that such effects cloud our understanding of genotype–phenotype associations. In considering these factors, some have proposed that we need an approach based on the prospective longitudinal study of infants at high-risk of developing autism (most commonly infant siblings in families with an older child already diagnosed with autism). Here the aim is to identify and study the early manifestations of the condition, less affected by atypical interactions with the social and physical environment. Studies of younger siblings of children with autism are motivated by the twentyfold increase in autism incidence in these groups with respect to the general population (Baird, Simonoff, Pickles, Chandler, & Loucas, 2006; Ozonoff, Young, Carter, & Messinger, 2011), making prospective studies feasible (Zwaigenbaum et al., 2007). Infants tend to be recruited in the first months of life and followed initially until 3 years of age, when a diagnosis can be made. A low-risk control group, composed of children that have no family history of autism, is typically followed in parallel. As simply having older siblings can affect development, control participants also have an older sibling. Around 20% of high-risk infants receive an autism diagnosis by their third birthday (Ozonoff et al., 2011). At-risk designs also allow the investigation of the broader autism phenotype (BAP, Bolton et al., 1994); sub-clinical traits or characteristics that are present at an elevated rate in families containing individuals with autism, with about 10–20% of high-risk infants developing sub-clinical ASD symptoms or other developmental problems (Messinger et al., 2013). The fast growing field of infant siblings (sibs) studies has been greatly motivated by the need to identify early reliable markers for this disorder, which would make possible earlier interventions. However, while markers are essential for early detection, a mechanistic understanding of autism emergence is crucial for designing efficient interventions. In addition, infant sibs studies offer the unique opportunity to tease apart different accounts about the origin of autism.

Like others before us (Pelphrey, Shultz, Hudac, & Vander Wyk, 2011), when discussing causes of autism, we choose to focus not on the genetic factors associated with this disorder but on the consequences they have on brain function. This is the level which best allows, for the time being, a mapping onto the spectrum of behavioral symptoms that are used to define and diagnose autism. A substantial number of neurobiological causes for autism have been proposed over the years, which can be roughly described as belonging to four lines of thought. Since autism is described as a disorder of social interaction and communication, some place the origin of autism within the category ‘disorders of the “social brain”’ (Adolphs, 2009). This set of hypotheses, one of the most widely accepted, branches out to focus on different components of the “social brain” (e.g., social orienting and sub-cortical structures, social motivation and reward networks or the processing of biological movement within the superior temporal sulcus). A second group of hypotheses proposes a domain-general developmental origin of autism. Many of these hypotheses still focus on circumscribed brain structures, for example the sensory cortices (Mottron, Dawson, Soulieres, Hubert, & Burack, 2006) or the frontal lobe (Hill, 2004). Another line of thought proposes that social and domain-general atypicalities influence additively the risk of developing autism symptoms (Happe, Ronald, & Plomin, 2006). Finally, inspired by increasing understanding of the genetic and molecular mechanisms involved in autism etiology, some autism researchers have suggested brain-wide neural impairments in factors such as long-distance connectivity (Kennedy & Courchesne, 2008), synaptic function and excitatory–inhibitory balance (Rubenstein & Merzenich, 2003) or mitochondrial function (Rossignol & Frye, 2012).

Since many of the infant at-risk studies are motivated by findings from adult populations and the theories emerging from these findings, we chose to organize our review accordingly. To address the key questions regarding the nature of the primary deficits in autism we will in turn assess the evidence for “social brain” impairments, and then for general-domain atypicalities like attentional control and sensory processing, in those infants who later develop autism. By *primary causes* we mean functional atypicalities at the level of neural networks or neural systems that are not the result of atypicalities within other systems and have not been compounded by atypical interaction with the environment. In some cases atypicalities within a neural system are proposed as primary only because no downstream causal factor has yet been described. This is a limitation that we acknowledge and will discuss later in the review.

Several studies have now described behavioral or neural markers that differentiate high-risk infants who will develop autism from those who will go on to develop typically. Because the interest is in autism before the onset of compensatory or compounding effects, we focus on studies of infants within the first two years of life, prior to the onset of overt clinical symptoms. By 2 years of age, behavioral symptoms like reductions in joint attention, atypical eye contact, reduced response to name, and language delays can lead to relatively stable diagnosis of autism in clinically referred samples (Woolfenden, Sarkozy, Ridley, & Williams, 2012). We then focus on studies with outcome diagnosis of autism or symptom severity at 2–3 years (see Jones, Gliga, Bedford, Charman, and Johnson (in press) for a more comprehensive review); group differences between high-risk infants and low-risk controls may reflect correlates of genetic risk factors, but can equally result from differences in family environment (i.e., growing up alongside a child with a developmental disorder), and are therefore more difficult to interpret. Twin studies of infants at risk for ASD will be required to tease apart genetic and environmental influences, in this case. This review of the literature will set the background for discussing existing models of autism development, including whether current evidence is more compatible with domain-specific and localized impairments or, in contrast, with models suggesting multiple independent hits (acting additively) or, alternatively, diffuse brain-wide atypicalities.

## “Social brain” hypotheses

### Reduced biases to orient to social information

Several authors have proposed that the social and communication delays observed in children with autism may result from reduced early orienting to social stimuli such as faces or spoken language, which in turn restricts the infant’s exposure to typical social interaction (Dawson et al., 2004; Gliga & Csibra, 2007). Decreased social exposure within one modality, as in deaf or blind children, is associated with difficulties with social interaction and understanding mental states (Parr, Dale, Shaffer, & Salt, 2010; Peterson & Siegal, 1999). One could expect that, if generalized across modalities, decreased orienting to conspecifics would have multiple and wide-ranging consequences on all aspects of development that require learning from people and about people. For example, gaze following may not develop because infants who are not drawn to faces and eyes do not notice the relationship between gaze direction and interesting objects in the world (Triesch, Teuscher, Deák, & Carlson, 2006).

A network of cortical and sub-cortical structures is involved in detecting and orienting towards social partners. The best characterized neural systems are those involved in orienting attention to faces. Together, the superior colliculus, pulvinar and amygdala use coarse spatial frequency information to orient attention to faces or eyes (Kennedy & Adolphs, 2010; Nguyen et al., 2013). Difficulties with orienting towards and engaging with people and their faces and voices have been frequently described in older children with autism (Chawarska, Macari, & Shic, 2012; Kikuchi, Senju, Tojo, & Osanai, 2009). Adults and toddlers with autism scan dynamic faces atypically, looking less at the eyes (Jones, Carr, & Klin, 2008; Klin & Jones, 2008; Klin, Jones, Schultz, & Volkmar, 2002). In addition, neuroimaging studies have identified atypicalities in the structure and function of the amygdala, in children with autism that are as young as two years of age (Nordahl et al., 2012; Swartz, Wiggins, Carrasco, Lord, & Monk, 2013). These findings have been highly influential in shaping theoretical and experimental approaches to autism in infancy.

**Fig. 1:**
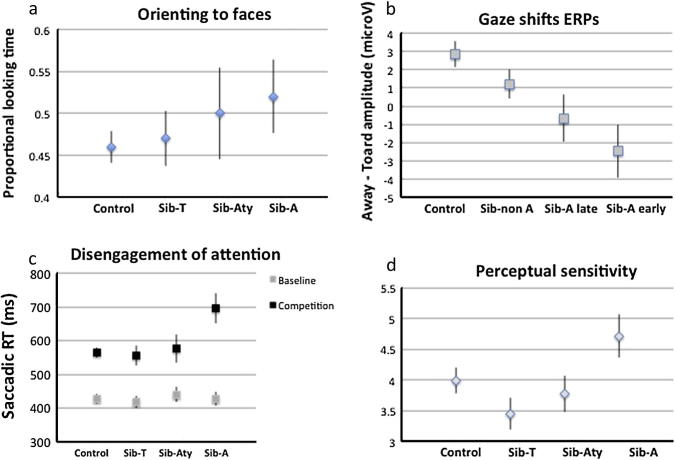
Different mappings between behavioral and brain markers and clinical outcome. Clinical classification of high-risk infants is carried out around 3 years of age and results generally in three sub-groups: a group of children that have developed autism (Sib-A), a group that have typical developmental outcomes (Sib-T), and a group that show some atypicality, generally developmental delays or sub-clinical autism symptoms (Sib-Aty). (a) At 7 months Sib-A orient and maintain attention to faces just as all the other groups do (Elsabbagh, Gliga, et al., 2012) (b) The difference between event-related responses to “towards” and “away” gaze looks atypical in all high-risk groups, but only Sib-A are significantly different from Controls (Elsabbagh, Mercure, et al., 2012); (c) Latency to re-orient from a central fixation to a peripheral target is much shorter in Sib-A, but looks typical in the other high-risk groups (Elsabbagh, Fernandes, et al., 2013) (d) Sensory sensitivity is highest in Sib-A but lowest in Sib-T. This could reflect a protective effect of decreased neural noise in somatosensory cortices (Clifford et al., 2013). This complex mapping between markers and clinical outcomes suggests multiple interacting developmental pathways are involved in the emergence of autism symptomatology. Bars represent standard error.

There is substantial evidence that neural systems that bias attention towards human faces or eyes are functional from the first months of life in typical development (Farroni, Mansfield, Lai, & Johnson, 2003). These mechanisms are actually most useful very early on, when limited attentional abilities make it difficult to select relevant information to attend to. It was suggested that orienting to faces and eyes in infancy relies on both sub-cortical and cortical networks, although evidence for the involvement of particular neural substrates is only indirect (Johnson, 2005). Thus, infants only a few days old preferentially orient to a schematic depiction of an upright face over an inverted face and to a face with open eyes over a face with closed eyes (Farroni, Csibra, Simion, & Johnson, 2002). If disruptions of these systems are involved in the development of autism, atypicalities should be identifiable within the first six months of life in infants who later develop autism (henceforth Sib-A). Surprisingly though, in the first year of life Sib-A are indistinguishable from typically developing infants on a variety of measures of early social orienting. Unlike older children and adults with autism (Jones et al., 2008; Klin & Jones, 2008; Klin et al., 2002), 6–9-month-old Sib-A show typical scanning of faces including a typical preference for looking at the eyes (Elsabbagh, Bedford, et al., 2013). Spontaneous orienting and engagement with static faces is typical at both 6 and 12 months (Elsabbagh, Gliga, et al., 2012; Fig. 1a). In a recent, densely sampled study of infants at risk, Sib-A oriented to the eyes more than their typically developing peers at 2 months of age. However, looking to the eyes decreases subsequently and becomes significantly lower than in controls at 24 months of age (Jones & Klin, 2013). Typical orienting to faces and eyes is measured in 6-months old Sib-A during live interaction (Ozonoff et al., 2010; Young, Merin, Rogers, & Ozonoff, 2009), but a decrease in orienting becomes apparent from 12 months (Ozonoff et al., 2010). One recent eye-tracking study of 6-month old infants did find decreased proportional time spent watching an actress’s face, but also less time looking at the screen in general. Since the study did not examine looking to stimuli with non-social content (Chawarska, Macari, & Shic, 2013), these results could potentially reflect general difficulties with maintaining attention to the screen rather than decreased social attention (see later on, when we discuss attentional control in Sib-A).

### Reduced social motivation

Decreased social motivation was recently proposed as an explanatory theory of autism (Chevallier, Kohls, Troiani, Brodkin, & Schultz, 2012). Being able to identify conspecifics in the environment is not sufficient; a lack of motivation to take part in social interaction, or to seek it when unavailable, can also result in decreased learning from and about people. Whilst sub-cortical orienting mechanisms are crucial during the first months of life, reward structures are involved in generating pro-social behavior throughout life. The region of the “social brain” involved in computing and associating reward value with various kinds of stimulation is the ventro-medial prefrontal cortex (i.e., the orbito-frontal and anterior cingulate areas; Rolls, 2000). In the case of social interaction, the orbito-frontal cortex computes social hierarchies and individual preferences (Azzi, Sirigu, & Duhamel, 2012). Together with sub-cortical structures like the amygdala and the insula, these cortical structures are believed to underlie general pro-social behavior (Rilling, King-Casas, & Sanfey, 2008). Children with autism rarely initiate social interaction and communication. Decreased initiation of joint attention (e.g., through pointing) is one of the most reliable early signs of autism (Dawson et al., 2004). A few studies have documented reduced activation of reward networks by social rewards in children and adults with autism (Zeeland et al., 2010, but see Ewing, Pellicano, & Rhodes, 2013 for evidence that children with autism put as much effort as typically developing children into seeing faces during a computerized task).

To show atypical social motivation in autism one should be able to measure “wanting” and “liking” social interaction in infants (Berridge, Robinson, & Aldridge, 2009). Older children and adults can overtly identify and communicate states of wanting and liking. In infants and other species these states need to be inferred from behavior and its consequences. For example, effortful approach toward and consumption of rewards is seen as a reflection of “wanting”. Simple orienting to faces in an experimental situation – often described as primitive motivated behavior – requires little effort from the infant. Although this is not evidence against reward system involvement in face orienting, it is also not strong evidence in support of it. “Liking” is not necessarily easier to identify in infancy. Emotional expressions of smiling or crying as well as physiological changes in heart rate, are used as indexes of well-being and pleasure but the exact relationship between these behaviors and the underlying subjective states of liking remains elusive.

The strongest (and most replicated) evidence for socially motivated behavior in infancy comes from reactions elicited by the sudden interruption in social interaction, known as the still face paradigm (Toda & Fogel, 1993; Weinberg & Tronick, 1996). To re-establish contact when the parent or an experimenter looks away or stops interacting with them, infants make considerable efforts, including smiling, gurgling, and vocalizing, which they stop when the experimenter returns. These could be seen as reflections of “wanting”. If the still face is maintained beyond a few seconds most infants show negative affect (dis-“liking”). Confirmatory evidence for reward network activation during infancy is still limited. Increased gamma-band activity was measured over frontal regions in 4-month-old infants while they were presented with a human face establishing mutual gaze (Grossmann, Farroni, Johnson, & Csibra, 2007), but this finding awaits replication using methods that have better spatial resolution. Using functional magnetic resonance imaging (fMRI) Blasi et al. (2011) measured increased insula and orbito-frontal activity in 3- to 7-month-old infants listening to sad voices. Measuring brain activity while the infant takes part in a still face paradigm is still not possible as because of the constraints imposed by MRI methodology – one needs to lie very still which can only happen if infants are asleep. Other methods allow brain measurement while the infants are awake (e.g. near-infrared spectroscopy, Lloyd-Fox et al., 2009) but cannot detect activity in “deep” structures, like the orbito-frontal cortex or the insula.

Behavioral responses to the ‘still face’ paradigm were measured in studies of high-risk infants, but were found to be typical at 6 months in Sib-A (Rozga et al., 2011; Young et al., 2009). Maternal reports of typical positive affect (i.e. smiling) during caretaking and interaction are also suggestive of typical reward system functioning, at least during the first 6 months of life (Clifford, Hudry, Elsabbagh, Charman, & Johnson, 2013). However, as we stated earlier on, it is still unclear whether smiling at this age reflects the influence of reward systems. In addition, the temperament questionnaire used by Clifford et al. (2013) does not differentiate between smiling during social interaction and smiling during non-social interaction. It is interesting to note, though, that mothers report a decrease in smiling from 12 months onwards in this study, around the same age at which a decrease in social orienting during interaction is observed (Ozonoff et al., 2010). Low infant positive affect and infant attentiveness to parent, recorded at 12-months during an episode of parent–child interaction, predict 3-year autism outcome (Wan et al., 2013). No such relationship was found between 6-month-old parent–child interaction measures and outcome.

In sum, current findings provide mixed support for a deficit in social orienting or social reward during the first months of life in those infants who develop autism symptoms. Orienting to people, and wanting and liking social interaction, have been measured in a variety of contexts; from highly controlled experimental studies to “real life” interaction. It is therefore unlikely the null results were a result of poor ecological validity. Rather, the possibility remains that the decreased social engagement observed at age of diagnosis (and by 12 months of life in Sib-A) is the developmental consequence of impairments in a different functional system during infancy. Decreased social orienting and motivation could, for example, be a *consequence* of difficulties in processing the incoming social information (e.g., in processing biological movement and actions), rather than their cause.

### Atypical processing of biological movement and actions

This set of hypotheses state that a primary factor in autism is not in the lack of social orienting or social motivation, but rather in processing of the information conveyed by human behavior. It was hypothesized that understanding and using gaze direction, emotional expressions or action goals requires specialized neural mechanisms that go beyond processing form or movement. Indeed, parts of the superior temporal cortex, together with other associative cortex regions the STS connects to, as for example the temporal–parietal junction, have been selectively associated with the processing of body and face movement that together are referred to as biological motion (Allison, Puce, & McCarthy, 2000; Kaiser, Shiffrar, & Pelphrey, 2012). These computations and their underlying neural substrates are atypical in autism. Children and adults with autism process bodily movement (Annaz, Remington, & Milne, 2009) and gaze (Leekam, Lopez, & Moore, 2000) atypically, and fMRI studies have shown that body or gaze movements produce less specialized activation of the STS (Zilbovicius et al., 2006, but also see evidence for general motion processing difficulties; Jackson et al., 2013). A lack of preference for upright over inverted point light displays of human action has also been documented in toddlers with autism (Klin, Lin, Gorrindo, Ramsay, & Jones, 2009).

To date, infant sibs studies have focused on gaze processing, with the aim of explaining later atypicalities in joint attention. Gaze perception develops gradually during the first year of life in typical development (Symons, Hains, & Muir, 1998), but rudimentary forms of gaze-following are present from days after birth (Farroni et al., 2003). Impairment in gaze processing networks in autism could therefore be measurable within the first months of life. In support of this hypothesis, event-related potentials (ERP) resulting from viewing changes in gaze direction showed that, unlike infants with later typical development, 6- to 9-month-old Sib-A did not differentiate between faces that shifted gaze away from faces that shifted gaze towards the viewer (Elsabbagh, Mercure, et al., 2012; Fig. 1b). For all groups, neural responses to faces differed from neural responses to control visual stimuli, suggesting that more basic aspects of face processing are intact early in life in those infants who develop autism symptoms. Behavioral measures of gaze following seem typical around 6 months of age in Sib-A, but impairments become apparent at the beginning of the second year, when Sib-A follow gaze but spend less time than controls looking at the gazed-at object (Bedford et al., 2012; Landa, Holman, & Garrett-Mayer, 2007).

It is still unknown whether difficulties with processing gaze measured at 6 months (Elsabbagh, Mercure, et al., 2012) reflect impaired STS functioning, or STS connectivity with other areas. Functional near-infrared spectroscopy (fNIRS) has recently been used to investigate cortical activity, including activity localizable to the temporal lobe, in 4- to 6-month-old infants. This series of studies have shown that in typical developing infants, temporal lobe activation is tuned to visual and auditory social stimuli (Lloyd-Fox et al., 2009). Ongoing work with this technique in high-risk infants (Lloyd-Fox et al., 2013) will help us understand the role of the STS in the emergence of autism symptoms.

In conclusion, there is some limited evidence that, at the age at which behavioral measures of social orienting and social motivation seem typical, Sib-A show atypical processing of one type of biological movement: gaze direction. Future studies will have to investigate whether it is gaze in particular, or any type of biological movement that is impaired early on in those children that develop autism symptoms. Interestingly, *longer* looking towards faces at 6 months of age, in high-risk participants, correlates with poor face recognition skills at 3 years of age (de Klerk, Gliga, Charman, Johnson, & The BASIS Team, 2014). This suggests that early face orienting (Jones & Klin, 2013) may also reflect atypical processing of faces. Processing of stimuli outside the visual modality, but which rely on STS functioning (e.g. voices, “social touch”), will also help describe the nature of these early difficulties. Since STS functioning relies on connectivity with a variety of sensory cortices and with higher-level structures within frontal and parietal areas, impairments in either sensory processing or connectivity could manifest as STS atypicalities. Another question that remains to be answered is whether difficulties with processing gaze direction can lead to the decrease in orienting to eyes and faces measured in the second year of life in Sib-A (Jones & Klin, 2013; Ozonoff et al., 2010). Just as stimuli associated with predictable reward become more salient in time (Sali, Anderson, & Yantis, 2012) so other stimuli (e.g., faces) could decrease their saliency if they are perceived as being unpredictably related with rewards. A variety of (and combinations of) social cues announce rewards in social interaction (e.g., gaze direction can indicate the location of desired food). An infant who has difficulties processing these cues might perceive them as unpredictable and therefore lose interest in them. This gradual avoidance of social interaction (and preference of repetitive, predictive aspects of the environment) may also be adaptive in providing the autistic brain with the type of information it can better process (Johnson, Jones, & Gliga, in press).

## General neuro-cognitive factors

### Reduced control of attention

Another set of hypotheses focus more on the non-social symptoms of autism such as rigid and repetitive behaviors, restrictive interests, and resistance to change (Leekam, Prior, & Uljarevic, 2011). These difficulties have been attributed in part to deficits in prefrontal cortical mechanisms of executive control (Lopez, Lincoln, Ozonoff, & Lai, 2005). In addition to explaining these non-social deficits, difficulties with executive control could underpin social interaction skills, like joint attention, because of the requirement for repeated and rapid shifts of attention between people and the objects they interact with (McEvoy, Rogers, & Pennington, 1993). It has also been suggested that good executive function may act as a protective factor across different developmental disorders (Johnson, 2012).

The ability to shift visual attention is a component of executive control skills and is associated with fronto-parietal modulation of sub-cortical saccade control mechanisms (Alvarez & Emory, 2006). Older children and adults with autism have difficulty in disengaging attention from a central fixation stimulus in attention competition tasks (Kawakubo et al., 2007; Landry & Bryson, 2004; but see Fischer, Koldewyn, Jiang, & Kanwisher, 2013 for contrasting findings). Interestingly though, the nature of the central fixation stimulus is important, as toddlers with autism take less time to disengage from a face (Chawarska, Klin, Paul, & Volkmar, 2007). This raises the possibility that differences in “engagement” with particular stimuli, and not general attention shifting, distinguish between neurotypical and autistic individuals. Many attention competition tasks that have shown disengagement difficulties use repeated and predictably moving central fixations (e.g. a spinning animation), which may be particularly “engaging” for individuals with autism.

Saccadic reaction times become faster in target competition tasks during the first year of life (Csibra, Tucker, Volein, & Johnson, 2000), possibly due to enhanced connectivity between fronto-parietal and visual areas (Elison et al., 2013). It is around 12–14 months of age that Sib-A show slower disengagement of attention than infants with other outcomes (Elsabbagh, Fernandes, et al., 2013; Zwaigenbaum et al., 2007; Fig. 1c). Changes between 7 and 14 months of age also predicts later diagnosis of autism (Elsabbagh, Fernandes, et al., 2013; Zwaigenbaum et al., 2005), suggesting that atypicalities of attention control might, in some cases, have their onset earlier, during the first year of life. Indeed, disengagement of attention at 6 months of age was slower in infants who met criteria for autism at 2 years of age (Elison, Paterson, et al., 2013) which suggests difficulties with attention shifting are apparent earlier in life in those infants whose symptoms of ASD are manifested already at around 2 years of age (compared to 3 years of age, as in the other studies). Interestingly, Elison, Paterson, et al. (2013) also found that slow disengagement correlates with fiber integrity in the splenium of the corpus callosum. Structural atypicalities have also been measured within projection pathways connecting frontal and parietal areas to posterior cortical areas in 6-month-old Sib-A (Wolff et al., 2012) but have not yet been related to saccadic reaction times.

**Fig. 2:**
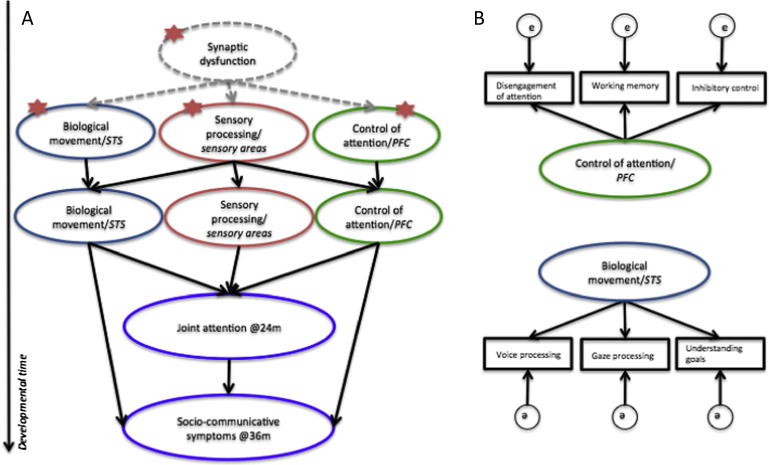
Possible models of how different neurocognitive systems contribute to the emergence of autism symptoms. In the example model shown in (A), early impairment in synaptic function within sensory cortices is likely to influence the development of biological movement processing (e.g. gaze direction) and of cognitive control skills. Alternatively, more generalized synaptic dysfunctions might affect the maturation and functioning of a variety of neural systems. Insults within any of these systems (marked as stars), early in development, can lead to difficulties in joint attention – one of the hallmarks of autism – which in turn can contribute to delays in social and communication skills. (B) To infer the involvement of a particular neuro-cognitive system in autism they can be modeled as latent variables underlying performance across a number of tasks (markers). ‘e’ represents additional sources of unmodeled variance.

In conclusion, as with the other hypotheses reviewed earlier, evidence for cognitive control difficulties comes only from a limited range of measurements (mainly of competition for visual attention). To clearly describe the mechanism leading to autism symptoms, future work will need to investigate performance in a wider range of attention control tasks (see Fig. 2b). Further evidence for frontal cortex structural or functional atypicalities during infancy, as well as evidence that attentional control early in infancy explains later difficulties with social interaction (e.g., gaze following) is also crucial to establish attentional control as a primary deficit in autism (but see later for evidence against this association). Alternatively, a better understanding of the conditions under which slower disengagement of attention is measured might point to alternative trajectories of cortical specialization (not for faces or biological movement but for predictable, mechanical stimuli; Johnson et al., in press).

### Atypical sensory processing

Reliable sensory processing is crucial for extracting regularities from the environment and for producing reliable motor outputs. Sensory problems, manifested as either hyper- or hypo-reactivity to stimulation, are prevalent in autism (Crane, Goddard, & Pring, 2009), and the critical role of sensory difficulties (Ben-Sasson, Soto, Martinez-Pedraza, & Carter, 2013) is recognized by their inclusion in the new DSM-5 (American Psychiatric Association, 2013) criteria for autism. It is proposed that increased noise in sensory systems during infancy may become compounded during subsequent development and particularly affect domains of perception and cognition that involve complex and dynamic events, such as those characteristic of understanding or taking part in social interaction. Detecting social stimuli in the environment, processing social information, or switching attention, could all suffer from atypical sensory input (Fig. 2a).

Evidence for altered sensory processing comes from studies showing that, for individuals with autism, auditory perception in silence is not superior to auditory perception in noise, as it is in controls (Russo, Zecker, Trommer, Chen, & Kraus, 2009). Imaging studies also show increased variability in neural responses to sensory stimulation. For example, studies in older participants with autism have reported increased intra-participant variability in electrophysiological (e.g., visual-evoked ERPs; Milne, 2011) or hemodynamic responses to different types of sensory stimulation (e.g., auditory, visual and tactile; Dinstein et al., 2012), and this variability was shown to correlate with the severity of autism symptoms (Dinstein et al., 2012). The mechanisms behind this increase in response variability are not known but we do know that during typical development, neural responses to sensory stimulation decrease in variability (Little, Thomas, & Letterman, 1999). During infancy, responses to familiar stimuli are also less variable than responses to novel stimuli, suggesting that a decrease in variability may reflect sensory learning and increased specialization of neural representations (Thomas et al., 1997).

Recent evidence suggests an early onset of sensory atypicalities in Sib-A. In one study, 7-month-old infants who later went on to develop autism were rated by their parents as more sensitive to low environmental stimulation than siblings that were typically developing (Clifford et al., 2013; Fig. 1d). These differences persisted at 12 months of age. In another prospective study, taking into account 12-month-olds’ sensory regulatory behaviors, in addition to social and communication behaviors, increased the ability of a parental-report questionnaire to predict later social and communication skills (Ben-Sasson & Carter, 2013).

Since somatosensory, and also visual or vestibular, feedback are needed for motor control (Osborne, Lisberger, & Bialek, 2005), sensory problems may also result in motor control problems. Investigations of motor skills in high-risk siblings have resulted in mixed findings. Six-month-old Sib-A showed more prominent head lag when lifted (Flanagan, Landa, Bhat, & Bauman, 2012) and delays in reaching motor milestones have been observed in a recent prospective study investigating posture development during the first year of life (Nickel, Thatcher, Keller, Wozniak, & Iverson, 2013). However, standardized measures of general gross motor abilities do not differentiate Sib-A from the other high-risk siblings earlier on (Landa, Gross, Stuart, & Faherty, 2013; Leonard, Elsabbagh, Hill, & The BASIS Team, 2013), with differences appearing only after 14 months of age (Landa & Garrett-Mayer, 2006).

More research is needed to understand the neural underpinnings of sensory and motor difficulties in autism. Other accounts of auditory hypersensitivity in autism, for example a relationship to low vagal activity, should be explored (Patriquin, Scarpa, Friedman, & Porges, 2013). It is also important to understand whether sensory and motor systems can be independently affected, as it is likely that difficulties with motor control arise independently of impaired somatosensory or proprioceptive input. Notwithstanding these limitations, the existing evidence for early onset of sensory hypersensitivity and motor atypicalities make this a promising avenue for future research.

## Explaining the diversity of early markers for autism

Infant sibs studies have been successful in identifying early markers that satisfy most proposed theoretical accounts of autism development, with the exception of social orienting and social motivation, which seem typical in Sib-A during the first year of life. Before their first birthday, those infants that later develop autism symptoms show atypicalities related to “social brain” functioning (at least as indexed by gaze processing), attentional control and sensory processing. There are two possible explanations for these findings: the variety of markers identified can reflect additive effects of independent factors or, alternatively, they can be manifestations of a common underlying mechanism that affects a great number of brain systems. We thus return to the last two theoretical views on the etiology of autism described previously to discuss how compatible they are with current findings from the infant sibs literature.

### Combined effects of multiple risk factors

With a few exceptions (Happe et al., 2006), theories of autism have been built on the premise of one necessary and sufficient initial deficit having knock-on effects on many other domains of cognitive development, leading eventually to the pervasive nature and phenotypic variability that are characteristic of the disorder. However, since a variety of genetic factors have been associated with autism diagnosis, each explaining less than 1% of ASD cases (Geschwind, 2011), a new view has emerged that autism phenotypic variability is a result of a combination of multiple genetic factors, none of which is necessary or sufficient for autism emergence (Plomin, Haworth, & Davis, 2009).

**Fig. 3:**
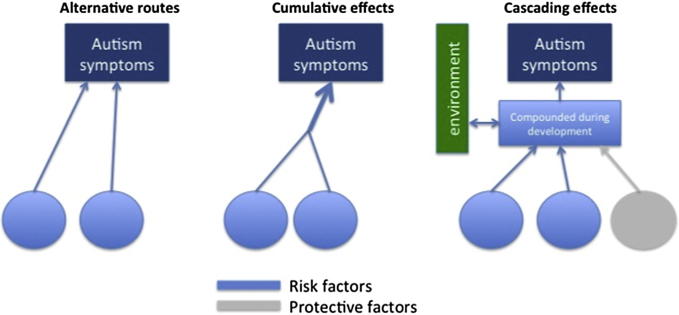
More than one developmental pathway could be involved in the emergence of autism symptoms. In the *Alternative routes* model impairments in independent developmental pathways can lead to autism (possibly to different sub-groups of individuals with autism). According to *Cumulative risk* hypotheses, two or more developmental pathways need to be atypical for autism to result. Developmental pathways are expected to interact in which case *Multiplicative* effects are expected. Protective factors (in grey) can lead to resilience in certain individuals. Risk and protective factors can be internal (genetically determined neuro-cognitive factors) or environmental. To tease apart between different models, a variety of neuro-cognitive and environmental factors (e.g. gaze following, attention disengagement, perceptual sensitivity, quality of caregiving) have to be assessed in the same individual and at different time points along development.

There are different ways in which independent risk factors can interact during development (Fig. 3). According to *cumulative* accounts of autism, the number of manifested atypicalities in neurocognitive systems during infancy will be related to the severity of the outcome. Different factors could contribute more or less to predicting an outcome. Additive effects of genetic risk markers were documented in schizophrenia (Purcell et al., 2009) and in ADHD (Jain et al., 2011). While cumulative models assume that the number of initial “hits” determines outcome, *multiplicative* models account for the dynamic nature of functional brain development by assuming that a few initial factors interact and amplify (or decrease) each other’s effects during development. Multiplicative effects of gene–gene interaction on brain function (Yacubian et al., 2007) and behavior (Lakatos et al., 2003), have been described. One should be reminded, nonetheless, that the multivariate models we described above are based on the premise that there is a unitary autism phenotype, varying only in severity. This assumption has been brought into question by evidence that different symptoms are correlated only weakly (Happe et al., 2006) and that phenotypic fractionation of the autistic population into sub-classes (e.g., language impaired or not or having sensory processing problems or not) could lead to better genetic linkage (Hu, 2012). This opens the possibility that there are *alternative* routes to autism that are segregated all the way through from the genetic to the clinical phenotype level (Fig. 3). Such a model has been suggested recently to explain phenotypic variability among patients with ADHD (de Zeeuw, Weusten, van Dijk, van Belle, & Durston, 2012). Of course, these under this scenario, one has to explain why sub-groups end up being classified together, clinically.

Whether or not developmental models will uncover alternative or convergent pathways depends also on the level at which the investigation is carried out. Neural systems (e.g., the “social brain”) have been proposed as a point of convergence of different atypical developmental pathways in autism (Pelphrey et al., 2011). Findings from infant at-risk literature are not compatible with impairments confined to the “social brain” although it remains possible that the “social brain” could be excessively affected by earlier distributed impairments. Moving a level down, to synaptic and neural network function, reveals other points of apparent convergence (see next section).

Although previous studies have demonstrated cumulative effects of behavioral risk markers in predicting autism outcome (e.g., Ben-Sasson & Carter, 2013), only one published study has directly compared different models of risk accumulation. Bedford et al. (2014) focused on gaze following (Bedford et al., 2012) and disengagement of attention (Elsabbagh, Fernandes, et al., 2013) measured at one year of age to show that they *independently* and *additively* predict clinical outcome. The different mappings of early markers onto clinical outcome groups (some differentiating Sib-A from all other groups, others distinguishing the whole at risk group, Fig. 1) are compatible with independent (additive or multiplicative) contributions to autism emergence. The independence of gaze following and disengagement of attention measures at 14 months, speaks against the hypothesis that poor attentional control could be a unique primary cause of autism, with subsequent cascading effects on gaze following (no relationship was found between disengagement of attention at 7 months and gaze following at 14 months, either; Bedford, 2012). Although the evidence for independent contributions in predicting later diagnosis is compatible with alternative causal pathways to autism, it remains possible that both attentional control and gaze processing difficulties result from a common, distributed underlying disturbance, but one that does not affect different neural systems in the same manner.

### Brain-wide neural atypicalities

The difficulty in identifying universal genetic factors in autism seems incompatible with the existence of *unique* factors (i.e., that would be necessary for any individual to manifest this disorder). Similar predictions can be made based on the evidence for independent contributions of different risk markers in predicting autism diagnosis. However, recent molecular biology findings suggest that large sets of ASD candidate genes contribute to molecular pathways that converge on a few functional networks (Hu, 2012; Voineagu et al., 2011), mainly involved with neuronal transcription regulation and synaptic plasticity (Parikshak et al., 2013). Some of these networks are particularly involved during pre-natal cortical development (Willsey et al., 2013) while others were associated with post-natal down-regulation of synaptic density (Tsai et al., 2013). Synaptic dysfunctions can lead to excitatory–inhibitory imbalance, which has also been proposed to have a causal role in autism (Rubenstein & Merzenich, 2003). Yizhar et al. (2011) have shown that increased neural excitability can lead to impairments in social interaction and cognitive functions in a mouse model of autism. Impairments in neuronal function are likely to also lead to disturbed short- and long-distance connectivity (Zatorre, Fields, & Johansen-Berg, 2012). Abnormal patterns of neural connectivity have indeed been associated with autism (Courchesne & Pierce, 2005; Geschwind & Levitt, 2007; Just, Keller, Malave, Kana, & Varma, 2012) and atypical (increased) connectivity was found in 6-month-old Sib-A (Wolff et al., 2012). Disturbed long-distance connectivity can in turn impact on many aspects of cognitive development. Increased connectivity between fronto-parietal and visual areas at 6 months is associated in typical development with slower disengagement of attention (Elison, Paterson, et al., 2013). Joint attention abilities at 9 months have been associated in typical development with increased frontolimbic circuit connectivity (Elison et al., 2013). Whether the increased connectivity in 6-month-old Sib-A predicts later difficulties with disengagement of attention or with joint attention in this population remains unknown. The causal link between excitatory/inhibitory imbalance, connectivity and behavioral atypicalities in Sib-A remains highly speculative. However, this is an exciting avenue for future research and one which may bridge the gap between human and animal models of autism. For example, using proton magnetic resonance spectroscopy, which can measure concentrations of glutamine and glutamate *in vivo* (Corrigan et al., 2013) one might shed light on whether excessive excitatory activity is present early in the development in Sib-A.

## Targeted early interventions for autism

Intervening in development is, finally, the only way in which *causal* developmental theories of autism can be validated (Bradley & Bryant, 1983). For this reason we briefly review the existing early interventions from the perspective of the developmental pathways they may be targeting. Not all of these interventions have yet been aimed at children with autism and only a few at infants at-risk for autism.

*Improving social information processing:* Joint attention training, which uses behavioral and developmental techniques to scaffold and enhance the child’s ability to share attention to objects and activities with adults, is effective for young children with autism (Charman, 2011; Dawson et al., 2010; Kasari, Gulsrud, Wong, Kwon, & Locke, 2010). Targeting component skills like gaze following as well as dyadic, interactive approaches (Wallace & Rogers, 2010) is proving a successful way to adapt such interventions for younger populations. Other interventions specifically target component social interactions skills such as imitation (Field, Sanders, & Nadel, 2001). Computer-based interventions targeted at the face-processing network in which children identify and interpret facial cues at increasing levels of difficulty show some benefit (Tanaka et al., 2010, 2012) and may prove a model for targeting intervention development to particular neurocognitive systems. Many of these interventions aim to increase the ability to process social information as well as motivation to attend to that kind of information.

*Improving cognitive control:* In typically developing pre-schoolers, cognitive control has been improved by training on increasingly difficult computer-based games requiring sustained attention, anticipation and discrimination (Rueda, Posner, & Rothbart, 2005) and by a tool-based preschool curriculum that includes intensive focus on control-promoting activities like self-regulatory private speech, dramatic play and memory and attention aids (Diamond, Barnett, Thomas, & Munro, 2007). Cognitive control (Tamm et al., 2008) and working memory (Klingberg, 2010) can also be improved by training in children with ADHD. Computer-based interventions that reward infants for sustained attention and cognitive flexibility increase cognitive control in typically developing infants (Wass, Porayska-Pomsta, & Johnson, 2011), and could thus have broadly beneficial impacts for younger siblings of children with autism who are at risk for a range of developmental difficulties.

*Improving sensory-motor processing:* Several types of intervention are available for improving sensory-motor processing in autism (Baranek, 2002). Sensory integration therapy and auditory integration therapy, which utilize a variety of anticipation, stimulation and calming techniques to modify the child’s response to sensory input, are widely used. However, there is presently little evidence of their efficacy (Lang et al., 2012). Although sensory stimulation techniques like massage therapy may have some efficacy in children with autism (Escalona, Field, Singer-Strunck, Cullen, & Hartshorn, 2001; Field et al., 1997). Early motor delays that are not autism-specific can also be addressed with general interventions designed for other populations (e.g., treadmill training for walking for infants with Down syndrome; Ulrich, Ulrich, Angulo-Kinzler, & Yun, 2001).

*Improving excitatory/inhibitory balance:* Transcranial magnetic stimulation (TMS) is able to up- or down-regulate targeted neural systems. Applied during various learning tasks (e.g., motor learning; Narayana et al., 2014 and number processing; Cohen Kadosh, Soskic, Iuculano, Kanai, & Walsh, 2010), TMS increases performance, possibly by stimulating local hebbian learning (i.e. enhancing the connections between cells active together). It is also suggested that TMS could modulate brain functional connectivity (Fox, Halko, Eldaief, & Pascual-Leone, 2012). More research into the long-term and side effects of this method, especially on the developing brain, is required before it can be used with infants at-risk.

The best-validated interventions for autism are intensive and combine developmental and behavioral approaches. It might be that they affect several of these neural and developmental systems simultaneously (Dawson et al., 2010). Recent innovative work has demonstrated that such interventions affect neural response to social stimuli (Dawson et al., 2012), although their effect on other neural systems such as attentional control, sensorimotor processing and neural connectivity has yet to be tested. Although success of some of these interventions would stand as evidence that the targeted mechanisms is causally involved in autism symptom emergence, they should only be seen as confirmatory evidence. Identifying robust early risk markers for this disorder, through studies of high-risk individuals, will allow the targeting of interventions to particular developmental causal pathways and to particular individuals, and thus decrease the cost of interventions.

## Future directions

Our review of the growing literature on early markers for autism points to a distributed impairment as a likely cause for this disorder. Research aimed at understanding whether the different markers identified converge at the level of a common underlying mechanism, or if they reflect factors acting independently, will benefit greatly from more sophisticated statistical modeling approaches and from innovative use of brain imaging. The great majority of published findings from infant at-risk studies have focused on isolated measures predicting later outcome. Convergent evidence from a variety of measures is necessary to delineate the nature of the underlying impairment. For example, if it is the case that STS functioning is impaired early in life in Sib-A, then gaze, action and voice processing are all expected to be atypical (see Fig. 2b). Statistical approaches such as factor analysis will better account for the noise intrinsic to each measure and increase predictive power. Predictive power might also be increased if protective factors, intrinsic or environmental, are taken into account. Such protective factors could manifest as predictors of typical outcomes in high-risk populations. A few studies have identified behavioral (Clifford et al., 2013) or neural signatures (Elsabbagh, Mercure, et al., 2012) that can differentiate unaffected individuals from their siblings with autism. For example, those high-risk infants who later achieve typical outcomes seek interaction (social or non-social) less than their high-risk peers that will go on to develop autism and also less than low-risk controls (Clifford et al., 2013). Decreased seeking might protect a brain that is struggling to process an excess of stimulation. Good executive control (Johnson, 2012) and genetic and cognitive factors specific to girls (Robinson, Lichtenstein, Anckarsäter, Happé, & Ronald, 2013) have also been proposed as protective factors against a variety of developmental perturbations/disorders and a range of pre-natal or early post-natal risk factors (e.g., parental age; Gardener, Spiegelman, & Buka, 2009; maternal pre-natal medication use; Rai et al., 2013; pre-natal stress; Ronald, Pennell, & Whitehouse, 2011) and protective factors (e.g., amount of physical contact used by depressed mothers; Sharp et al., 2012) have been shown to modulate the effects of gene expression, accentuating or diminishing the severity of later manifested clinical symptoms. Much more work is required to understand how protective and risk factors interact. For example, some factors may protect against specific risks (e.g., good language skills in girls diminishing the impact of atypical social interaction) whilst others may have wider impact (e.g., executive functioning, Johnson, 2012).

A better understanding of the relationship between structural and functional brain development during early infancy, and of the relationship between individual differences in brain and cognitive development, is also essential. In the near future it will become possible to use concurrently methods with good spatial resolution (e.g., NIRS) and temporal resolution (e.g., EEG) and to integrate functional information with structural information measured with MRI while infants are asleep. More than just a technological feat, this convergence of methods is theoretically important because it will allow researchers to put to the test localized (domain-specific or domain-general impairments) and brain-wide theories of autism development. To arbitrate between different hypotheses one may have to look at even earlier development. The great majority of sibs findings to date come from infants aged 6 months and over. Given the likely involvement of pre-natal and post-natal synaptic functioning in the etiology of this disorder, neural atypicalities and their phenotypic markers might be measurable at younger ages, and potentially even before birth. Behavioral measures of fetal sensory reactivity (Hepper, Dornan, & Lynch, 2012) and in utero brain imaging methods (Habas et al., 2012) provide possibilities for investigating autism even earlier during development. Some of the identified post-natal markers for autism that are now proposed as primary causes for this disorder, may turn out to be secondary effects of transitory pre-natal neural impairments (Johnson et al., in press).

Future studies will also have to address whether the identified developmental pathways are specifically involved in autism emergence as well as universally involved in explaining autism beyond high-risk populations. The high rate of co-occurrence between autism and other neurodevelopmental disorders, such as ADHD (Simonoff et al., 2008), suggests that there may be common risk factors. Aspects of atypical cognitive control and higher variability in sensory-motor processing have been found in older diagnosed children and adults with ADHD (Cortese et al., 2012), whereas poor emotion recognition has been shown to be specific to ASD (whether comorbid with ADHD or not) and is not found in individuals with ADHD only (van der Meer et al., 2012). In contrast to the rapidly accumulating body of work in the autism field, the concept of a ‘broader phenotype’ and the utilization of a familial high-risk design have only just emerged in the ADHD field (Gurevitz, Geva, Varon, & Leitner, 2012) and it will be several years before enough evidence has accumulated to assess the specificity of risk markers across neurodevelopmental outcomes (e.g., autism in isolation, ADHD in isolation, autism with ADHD). It is also important to make sure the markers identified in infant sibs studies can also predict autism in the general population. Markers that have showed high predictive power (e.g., Jones & Klin, 2013) may not be sufficient in predicting autism outside high-risk populations. Genetic factors associated with inherited autism (the case of Sib-A) are believed to differ from de novo mutations, leading to more severe phenotypes, incompatible with inter-generational transmission (Ronemus, Iossifov, Levy, & Wigler, 2014). On the other hand, social environmental factors, such as the presence of autism traits in siblings or other members of the family, might affect social interaction and in turn phenotypic expression of genetic risk factors in Sib-A, but not in sporadic cases of autism. It is therefore possible that risk markers identified in infant sibs will not be good predictors of autism in cases of de novo mutations.

Finally, understanding the primary biological causes of autism will further benefit from developmental work on mouse models, in which one can test the effect of localized or brain-wide neural atypicalities at different points in development (Yizhar et al., 2011). The focus on autism as a “social brain” disorder has often led to criticism of animal models that could not truly capture the social-and communicative deficits found in humans. In contrast, sensory reactivity and attentional control can be measured in a similar way in rodents and humans. For example, event-related potentials have been used to study habituation to sensory stimulation and orientation to novel stimuli in infants at-risk for autism (Guiraud et al., 2011) and in mice (Imada, Morris, & Wiest, 2012). More importantly, developmental mouse models will be crucial for demonstrating causality between neural and behavioral markers of autism (Delorme et al., 2013).

## Conclusions

We reviewed here evidence for brain-wide atypicalities during the early development of those infants who go on to develop autism. These atypicalities go beyond the “social brain” to encompass sensory processing and attentional control. As such, current evidence strongly supports distributed (brain-wide) factors involved in autism pathology. Whether a unique mechanism (e.g., synaptic dysfunction) is responsible for distributing impairments across functional systems, or a few independent factors act additively to lead to the emergence of autism, remains an open and pressing question. Answering this question should be the main focus of developmental autism research.
